# De-Escalating Anticancer Treatment: Watch Your Step

**DOI:** 10.3390/cancers17152474

**Published:** 2025-07-26

**Authors:** Jean-Marc Ferrero, Rym Bouriga, Jocelyn Gal, Gérard Milano

**Affiliations:** 1 Department of Medical Oncology, Antoine Lacassagne Center, University Côte d’Azur, 33 Avenue de Valombrose, 06189 Nice, France; jean-marc.ferrero@nice.unicancer.fr; 2 Medical Oncologist, Park Imperial Clinic, 28 Boulevard du Tzarewitch, 06000 Nice, France; 3 Department of Epidemiology and Biostatistics, Antoine Lacassagne Center, University Côte d’Azur, 33 Avenue de Valombrose, 06189 Nice, France; jocelyn.gal@nice.unicancer.fr; 4 Antoine Lacassagne Center, University Côte d’Azur, 33 Avenue de Valombrose, 06189 Nice, France

**Keywords:** cancer treatment, de-escalation, precision medicine, clinical trials

## Abstract

**Simple Summary:**

Cancer treatment has long operated under the principle that “more is better,” favoring aggressive approaches despite their side effects. With the advent of personalized medicine, however, there is growing support for treatment de-escalation—strategies that reduce treatment intensity without compromising efficacy. This paper examines how such approaches are being implemented across several cancer types, notably in breast cancer but also in colorectal, head and neck, ovarian, lung, and prostate cancers. Methods like dose reduction, shorter treatment duration, and simplified regimens have demonstrated the ability to lessen toxicity while maintaining outcomes. Emerging diagnostic tools, including genomic profiling and liquid biopsies (in particular, circulating tumoral DNA), enable better patient selection for de-escalation. While the early results are promising, further prospective studies are essential to validate these strategies and support their integration into routine oncology practice.

**Abstract:**

The concept of “more is better” has long dominated cancer treatment, emphasizing aggressive therapies despite their toxicity. However, the rise of personalized medicine has fostered treatment de-escalation strategies aimed at minimizing toxicity, improving quality of life, and reducing costs. This position paper highlights key applications of de-escalation in medical oncology, with a primary focus on breast cancer and notable examples in colorectal, head and neck, ovarian, lung, and prostate cancers. Various approaches, including dose reduction, treatment duration shortening, and regimen optimization, have demonstrated efficacy without compromising clinical outcomes. Advances in molecular diagnostics, such as Oncotype Dx in breast cancer and circulating tumor DNA (ctDNA) analysis in colorectal cancer, have facilitated patient selection for de-escalation. While these strategies present promising results, challenges remain, particularly in balancing treatment intensity with oncologic control. The review underscores the need for further prospective trials to refine de-escalation approaches and ensure their safe integration into standard oncologic care.

## 1. Background

“More is better” has long been a guiding principle in cancer treatment. The prevailing belief was that cancer management could be achieved through aggressive, intensive therapies, despite their often severe impact on patient tolerance. However, the progressive introduction of personalized medicine has led to the emergence of the concept of therapeutic de-escalation. It must be underlined that many factors other than precision medicine can contribute to the emergence of the concept of therapeutic de-escalation. They include better trials with optimal patient follow-up; better treatments; and better diagnosis, imaging, and pathology. One of the earliest examples of treatment de-escalation was in breast cancer management, where mastectomy was replaced by breast-conserving surgery plus irradiation. Several factors have driven the development of this approach. The primary motivation was to improve patients’ quality of life by reducing treatment-related toxicity. Another key reason was to minimize inherent toxicities and mitigate the long-term sequelae associated with anti-cancer therapies. Additionally, therapeutic de-escalation aims to reduce the significant costs linked to often-expensive cancer treatments. From a practical standpoint, treatment de-escalation can take several forms, including dose reduction, a decrease in the number of administered drugs, a shortened treatment duration, and the optimization of therapeutic indications [1].

Certain drugs like fluoropyrimidines may exhibit severe adverse reactions, which can be predicted on the basis of phenotyping or genotyping of key enzymes of their metabolism. This is the case for dihydropyrimidine dehydrogenase (DPD), and some (rare) patients at risk with identified DPD abnormalities may benefit from a complete omission of fluoropyrimidines, of which DPD is the key enzyme for catabolism [2]. The lowest step of treatment de-escalation strategy is reached in such a case. It is clear that trials investigating the impact of fully omitting a drug should be prioritized in terms of benefits in the quality of life and the treatment burden, including costs [1].

The objective of this position paper is not to provide an exhaustive review of cancer treatment de-escalation, but rather, to highlight important applications in medical oncology. While we emphasize breast cancer as a central example because of the clinically proven contribution of a biologically-based treatment de-escalation, we also discuss other tumor types including colorectal, head and neck, ovarian, and prostate cancers where notable advances in treatment de-escalation have been reported. Given that the clinical impact of therapeutic de-escalation may vary between neoadjuvant, adjuvant, and metastatic settings, special attention is paid to these specific contexts.

Recognizing that treatment de-escalation is not without limitations, part of this paper will explore the challenges and future perspectives of this strategy, aiming to delineate the boundaries between therapeutic reality and theoretical potential.

## 2. De-Escalation in the Clinical Reality

The continuous introduction of prognostic biomarkers appears to be of prime importance for optimally managing the strategy of therapeutic de-escalation. Several tumoral localizations, as described below, represent a concrete illustration, although not exclusive, of how the application of tumor biomarkers can fuel the driving of therapeutic de-escalation; among the family of tumor biomarkers at disposal to conduct treatment de-escalation there is the emergence of circulating tumor DNA (ctDNA) [3]. CtDNA measurement still needs, however, continuous technical improvements while it remains that its analysis becomes of paramount importance, as pointed out through numerous recent studies [4].

### 2.1. Breast Cancer

Breast cancer that is positive for estradiol receptors (ER+) and negative for HER2 expression (HER2−) is the most common form of the disease. One of the key challenges in its therapeutic management is identifying which patients will benefit from adjuvant chemotherapy. A major contribution to answering this question comes from the TAILORx prospective trial, which included more than 10,000 patients. This study was based on a tumor composite score incorporating 21 different biological variables related to proliferation, invasion, and hormonal response [5]. Considering an intermediary score (RS between 11 and 25), it was put into evidence that patients receiving, in an adjuvant setting, hormonotherapy alone had a similar evolution to patients treated with a hormonotherapy–chemotherapy association. Thus, it followed that the application of the Oncotype Dx test was beneficial in order to ensure a therapeutic decision concerning the application or not of chemotherapy for T2 tumors. The usefulness of tumor scoring with the Oncotype Dx test was strengthened following the results of the RxPONDER study questioning the benefit or lack thereof of an adjuvant chemotherapy applied to node-positive patients (pN1) with a Oncotype score between 0 and 25 [6]. The study clearly demonstrated that for patients over 50 years old with a score below 25, chemotherapy did not provide a significant benefit. This may serve as a concrete example of treatment de-escalation based on an objective and easily accessible quantitative tool.

HER2-positive breast cancer is traditionally treated in the adjuvant setting with a combination of chemotherapy and trastuzumab. A meta-analysis by Chen and co-workers [7] pointed out that a one-year treatment by trastuzumab brought a significant advantage in terms of disease-free survival. However, the meta-analysis by Earl and co-workers [8] indicated a non-significant decrease in survival when comparing 6 months to 1 year of trastuzumab in the adjuvant setting. The intensity of chemotherapeutic treatment has also been reconsidered in breast cancer. For instance, the study by Tolaney and co-workers has revealed that the application of anthracyclines was not indispensable for stage I breast cancer patients [9]. The regimen combined paclitaxel with trastuzumab for four cycles. The resulting event-free survival at three years was 98.7%, with a notably low incidence of cardiac insufficiency at 0.5%. Importantly, these findings have led to the adoption of this regimen in the therapeutic management of small breast tumors without nodal involvement.

### 2.2. Colorectal Cancer

In stage III colorectal cancer, standard adjuvant chemotherapy consists of six months of FOLFOX (5-fluorouracil, leucovorin, and oxaliplatin) or CAPOX (capecitabine and oxaliplatin). However, for patients with low-risk disease (T1–T3, N1), a three-month duration may be as effective as six months in terms of disease-free survival (DFS) [10]. However, the choice of regimen is crucial when considering this de-escalation, as subgroup analyses revealed that the non-inferiority of the three-month therapy was more pronounced with CAPOX than with FOLFOX [11]. In contrast, patients with high-risk disease (T4, N2) were more likely to benefit from the full six-month course of adjuvant chemotherapy, highlighting the importance of personalizing treatment decisions. A recent report by Gallois and co-workers [12] indicated that a combination between validated prognostic biomarkers complemented with emerging tumoral and microenvironment data could improve the prognostication for stage III colon cancers. On these bases, it was interesting to learn that only intermediate and high-risk groups could draw an objective benefit from 6 months of adjuvant therapy compared with 3 months [12].

Detecting minimal residual disease through the analysis of circulating tumor DNA (ctDNA) may improve the assessment of cancer recurrence risk and help distinguish patients who can safely avoid additional treatment from those who would benefit from adjuvant chemotherapy [13,14]. Conversely, for advanced colorectal cancer patients with the BRAF-V6000E mutation, where the therapeutic standard is an association of a RAF inhibitor plus an EGFR inhibitor [15], there is the beneficial possibility to escalate treatment with the addition of an MEK inhibitor when the plasmatic BRAF-V6000E allele fraction is high [16]. There is, however, a concrete limitation to the ctDNA application as concerns the analytical sensitivity of its measurement. For instance, in low-risk stage II colon cancer, ctDNA analysis could not be realistically applied due to the exceedingly low levels of this tumor-related parameter [17].

The use of treatment personalization factors such as ctDNA measurement is of prime importance, but it is not mandatory for conducting treatment de-escalation in digestive cancers. For instance, in the management of metastatic colorectal cancer, clinical guidelines support de-escalation approaches, including maintenance therapy, “stop-and-go” strategies, and drug holidays, without necessarily requiring a personalized approach [18].

### 2.3. Lung Cancer

The value of ctDNA in guiding treatment de-escalation was also prospectively evaluated in patients with advanced (stage III–IV) non-small-cell lung cancer (NSCLC) treated with tyrosine kinase inhibitors (TKIs). When imaging revealed no residual lesions, and after local consolidative therapy (LCT) for both primary and metastatic tumors, if ctDNA was not detectable, TKI treatment could be safely reduced or discontinued, resulting in no disease progression while reducing side effects [19]. In a recent review by Remon J et al., NSCLC patients exhibiting a complete or durable partial response to immune checkpoint blockers (ICBs) may benefit from a reduction in treatment dose, duration, and frequency, leading to diminished immune-related toxicities while maintaining efficacy [20]. Similarly, in stage III NSCLC, following concurrent chemoradiotherapy, patients with low-risk profiles or strong initial responses may benefit from less than one year of adjuvant durvalumab without compromising their outcomes [21].

### 2.4. Head and Neck Cancer

The success of radiotherapy–chemotherapy combinations in locally advanced head and neck cancers is well established [22]. A marked improvement is noticeable in the clinical outcomes for HPV-associated head and neck cancer. However, this therapeutic benefit is often tempered by deleterious treatment-related side effects. This context has spurred interest in the development of de-escalated therapeutic strategies for this tumor type with the goal of improving oncologic outcomes while reducing short- and long-term toxicity [23]. Overall, de-intensification strategies focus on replacing, reducing, or even omitting chemotherapy [23]. At present, however, there is an ongoing controversy regarding the optimal way to de-escalate treatment intensity, especially in HPV-positive head and neck cancer. A potential answer to this question may come from the recently published NRG-HN005 trial [24]. The purpose of the study was to prospectively compare the clinical results of definitive radiotherapy with or without systemic therapy in p16-positive head and neck cancer patients (444 patients, 2009 to 2022). The authors established a multivariate score (risk model) based on demographic, tumor, and biological characteristics. Overall, the findings showed that identifying patients with a relatively lower risk for cancer events could guide de-intensification strategies. Thus, the application of predictors of relative risk appears as an interesting option to individualizing de-escalation approaches in head and neck cancer.

### 2.5. Ovarian Cancer

In the management of advanced ovarian cancer, the introduction of poly (ADP-ribose) polymerase (PARP) inhibitors (PARPis) has ushered in a new era of treatment. The marked improvement in overall survival due to the application of PARPis highlights the possibility of minimizing the toxicity burden while maintaining oncologic control through the adoption of de-escalated treatment paradigms [25]. Properly selected patients with BRCA-mutated and/or homologous recombination-deficient tumors may be the right candidates for such de-escalated approaches. For instance, it is reasonable to consider that these patients could maintain a good prognosis while reducing the intensity of adjuvant chemotherapy after the complete surgical removal of bulky tumors. It is clear that alternatives, such as platinum salts and taxanes used in the adjuvant setting, can cause cumulative, dose-dependent, and long-term neurotoxicity, among other toxicities. The feasibility and safety of de-escalated approaches in ovarian cancer was recently reported with an overview of several ongoing promising clinical trials [25].

### 2.6. Prostate Cancer

Over the last decade, the systemic treatment of metastatic prostate cancer has drastically changed [26]. More than 90% of prostate cancer patients respond to anti-androgen treatment, either through surgical or medical castration. However, cancer cells can become resistant to castration through multiple mechanisms, including androgen receptor amplification or mutation, the production of ligand-independent splice variants, and androgen production by adrenal or cancer cells [27]. This multiplicity of resistance factors has led to the definition of therapeutic strategies combining different approaches such as those illustrated by the recent PEACE-1 trial, which showed the benefits of combining abiraterone with chemo-hormonal therapy [28]. On the other hand, while benefits in overall survival are observed in all patients, it remains uncertain whether the triplet therapy-combining androgen receptor signaling inhibitors, androgen deprivation therapy, and docetaxel should be the standard of care for all patients with metastatic castration-sensitive prostate cancer (mCSPC) [26]. This view opens the prospect of treatment de-escalation in prostate cancer. Prognostic models based on clinical variables can stratify mCSPC to a certain extent, but increasingly used tools from next-generation sequencing technologies, as well as genomic and transcriptomic data, are likely to more faithfully guide treatment strategies for treatment de-escalation [29]. The need to individualize therapy in the management of prostate cancer, underpinning the decision to de-escalate treatment or not, still largely depends on the efficacy and duration of hormonal therapy, including androgen receptor and signaling inhibitors. The introduction of omics tools in this context will certainly improve treatment adjustments on an individual basis. While randomized trials have been extensively applied in the field of radiotherapy dose escalation in prostate cancer [30], a similar approach would be welcome in the medical treatment armamentarium for prostate cancer, rigorously evaluating the benefits or drawbacks of adjusting treatment intensity based on objective and measurable predictive factors.

## 3. Treatment De-Escalation—Limits of the Theory

A systematic review and meta-analysis re-evaluated the impact of relative dose intensity (RDI) on survival for patients with solid tumors excluding those treated with adjuvant chemotherapy [31]. This retrospective analysis was based on the criteria of RDI, which is the ratio of the delivered dose intensity (mg/m^2^ per week) to the planned dose intensity for a given chemotherapy protocol. The included studies covered carboplatin-based chemotherapy in ovarian, breast, or non-small-cell lung cancer as well as FOLFOX- or FOLFIRI-based chemotherapy regimens in colorectal and pancreatic cancer. Results showed that shorter overall survival was associated with RDI < 80% versus >80% for carboplatin-based studies and with RDI < 85% versus > 85% for FOLFOX or FOLFIRI chemotherapy protocols. Among the multiple factors contributing to the reduction in RDI, hematological toxicity, many forms of which can be preventable, were identified. The authors concluded that maintaining higher RDI through the effective management of toxicities, irrespective of treatment modalities, would benefit survival for patients with advanced solid tumors. Overall, this meta-analysis implies that some caution should be taken regarding the current enthusiasm for de-escalating treatment intensity. This view is further supported by a recent report on the impact of anthracyclines in high-genomic-risk, node-negative, HR+, HER2-negative breast cancer [32]. This study showed that, after controlling for age, grade, tumor size, and HR status, the application of an anthracycline–taxane regimen in patients with a recurrence score (RS) ≥ 31 on the Oncotype DX test resulted in a significantly improved 5-year survival.

What has been reported above for some major chemotherapeutic drugs, where a direct link exists between tumor cell cytoreduction and the intensity of drug exposure, may not necessarily apply to anticancer agents with mechanisms of action not based on direct cytotoxicity. For instance, Op’t Hoog and coworkers [33] retrospectively evaluated the rationale and validation of dose selection for anticancer drugs approved by US and European health agencies from 2020 to 2023, most of which were targeted therapies and immune-modulating treatments. The analysis revealed that 65% of the 31 registered agents were potential candidates for dose optimization, either by reducing the dose (32%) or adjusting the dosage regimen (32%). To illustrate the point, we now have CDK4/6 inhibitors in the treatment of breast cancer. The main results arising from the MONARCHe and NATALEE trials [34,35], where abemaciclib and ribociclib were combined with anti-aromatases in HR-positive, high-risk breast cancer, demonstrated a significant improvement in overall survival with the drug combination. In this particular situation of high-risk breast cancer, it is clear that more treatment leads to better disease outcomes.

The case of immune checkpoint inhibitors (ICIs) is of prime importance in the general context of treatment de-escalation. A main reason for this lies in the projected global expenditure on ICIs, which is expected to sharply increase from 2021 to 2026 (from over USD 24 billion to USD 46 billion) [36]. This medico-economic context led Wesevich and coworkers to claim interventional pharmacoeconomics, primarily based on dose reduction and less-frequent dosing, as a concrete opportunity to reduce both the economic burden and safety concerns associated with ICIs [37]. This view may, however, be down-tuned in light of warnings regarding the potential reduction in the duration of treatment with ICIs. Concretely, it has been reported that the rate of complete response to ICIs in mismatch repair-deficient, locally advanced colorectal cancers is seemingly proportional to the duration of neoadjuvant therapy [38]. In this category of patients, the outcomes of the NICHE-2 trial are particularly impressive, with almost 70% of pathological complete response following a combination of two ICIs in a neoadjuvant setting [20,39]. This raises the question of a possible tumor immune ablation with organ preservation and the potential advantage of prolonging neoadjuvant treatment with ICIs to achieve the best possible response. Thus, these data do not support the necessity of de-escalation for ICI-based treatments. However, these findings do not preclude the need for further clinical exploration designed to establish the relative treatment heaviness conferred by ICIs, taken alone or in combination with chemotherapy [40], and also the optimal duration for ICI-based therapy, as particularly explored in lung cancer [41]. In this respect, the recent clinical study reported by Cascone and coworkers is particularly illustrative. The authors found that the use of no more than three ICI-based neoadjuvant cycles appears optimal in the management of non-small-cell lung cancer [42].

## 4. Final Remarks and Prospects

Considering the above-mentioned tumor locations, it globally appears that treatment de-escalation is a clinically rewarding approach, providing a good balance between safety and efficacy. This remains true whether or not the de-escalation is personalized, and more specifically, when it is guided by the use of tumor-related molecular markers such as the ctDNA highlighted herein (Table 1). In line with this consideration, recent data in the management of triple-negative breast cancer suggest that chemotherapy-free immunotherapy regimens could represent a therapeutic option in the neoadjuvant setting, subject to the required careful patient selection based on baseline immune marker expression [43]. In this context of biologically guided treatment de-escalation, the ongoing ETNA (Early Triple Negative breAst) controlled trial [44] in T1b and T1c triple-negative breast cancer should be mentioned, as it evaluates a de-escalation strategy based on the intratumoral quantification of tumor-infiltrating lymphocytes.

There are compelling reasons to expand the scope of predictive factors beyond the tumor itself by addressing the full range of patient characteristics. For example, variability in patient drug pharmacokinetics (PK) and pharmacodynamics (PD) data logically leads to pharmacokinetically adapted dose prescriptions, which allow not only for de-escalation but also for potential dose escalation, depending on the drug in question. This PK–PD approach has been thoroughly investigated by our group, particularly in relation to fluoropyrimidine (FU)-based treatment [51]. The cost-effectiveness of such a PK-guided FU-based chemotherapy has been firmly established [52]. Constitutional phenotyping, as in the case of UGT1A1 and DPD, can also be particularly useful. This has been demonstrated in gastrointestinal cancer, where a pretreatment application of DPYD- and UGT1A1-guided therapy was shown to increase safety and reduce hospitalization duration and related costs with the clinical benefit not affected [2]. More generally, taking into account the pharmacological rationale of treatment regimens may confer significant economic impact [53].

At the forefront of optimizing dose selection in oncology is the FDA initiative, “Project Optimus” [54]. In this project, data were collected from 50 oncology programs between 2019 and 2023 based on new FDA drug applications. The goal was to produce model-predicted outcomes using multisource data covering efficacy, safety, and PK–PD results. The findings revealed that an optimal benefit–risk ratio was consistently observed at PK exposures close to the recommended doses. More broadly, this FDA initiative could serve as a robust tool for assisting in the optimal dose identification for oncology drugs by balancing therapeutic benefits with risks [55].

## 5. Conclusions

The prevailing belief that more is better as a guiding principle in cancer treatment is based on the general principle that cancer management can be achieved through aggressive and intensive therapies at the expense of patient safety. However, the progressive improvement of personalized medicine has led to the appearance of the notion of therapeutic de-escalation.

The present review article emphasizes breast cancer as a central example of cancer treatment de-escalation, but other tumor types were also taken into consideration, including colorectal, head and neck, ovarian, and prostate cancers, where significant advances in treatment de-escalation have been pointed out.

All the covered examples constitute a concrete illustration of how the application of tumor biomarkers can fuel the management of therapeutic de-escalation. Among the family of tumor biomarkers under active consideration to concretely conduct treatment de-escalation, there is the emergence of circulating tumor DNA (ctDNA). CtDNA measurement still needs continuous improvements, but the clinical benefits of its analysis have been largely demonstrated in the management of treatment de-escalation.

Finally, and because the impact of treatment de-escalation is of prime importance for the patient, patient thoughts and quality of life, along with shared decision-making processes, it appears clearly that a careful involvement of patients and even the public needs to occur before planning a trial aimed at optimizing the use of drugs [1], especially in the case of treatment de-escalation. Such a contribution will contribute to the willingness to perform high-quality, large, optimal drug use trials in order to obtain a profound and durable impact on patients and societies [1].

A personalized approach to drug de-escalation, as well as drug escalation in less frequent cases, should consider not only tumor-related biological factors but also patient- and pharmacologically related characteristics [1]. This includes not only pharmacokinetic (PK) behavior but also the pharmacogenetic profile, both of which can significantly influence drug exposure and impact pharmacodynamics (PD). Deep learning and machine learning models can no doubt provide a particularly effective solution for managing the multifactorial factors involved, enabling a rationale for selecting the optimal treatment de-escalation strategy [56]. This opens up the possibility of a new step forward in treatment personalization and potential de-escalation, with applications guided by rigorously conducted clinical evaluations.

## Figures and Tables

**Table 1:** Treatment de-escalation highlighting key therapeutic findings.

Tumor Location	De-Escalation Principle	Personalized Approach	Main Results	References
**Breast Cancer (HER2−, ER+)**	Application of the Oncotype Dx in deciding adjuvant chemotherapy	Yes (Oncotype Dx score)	- The TAILORx trial showed that patients with an intermediate recurrence score (RS 11–25) had similar outcomes with hormonotherapy alone compared with combined hormonotherapy and chemotherapy. - For node-positive patients, the RxPONDER study indicated no significant benefit of chemotherapy for patients aged > 50 years with a score < 25.	[5,6]
**Breast Cancer (HER2+)**	Reduction in the adjuvant treatment duration with trastuzumab Adjustment of chemotherapy intensity	No	- A meta-analysis showed a non-significant difference in survival between 6 months and 1 year of adjuvant trastuzumab. - In stage I cancer, anthracycline-free therapy showed 98.7% 3-year event-free survival and low cardiac toxicity.	[7,8,9]
**Colorectal Cancer** **(Stage II)**	Identify patients who can safely avoid adjuvant chemotherapy Detect minimal residual disease using ctDNA to assess the risk of recurrence	Yes (ctDNA)	- High-risk stage II colon cancer patients benefit significantly from adjuvant chemotherapy, with a 5-year survival improvement (84.9% vs. 66.3%). - Elderly patients (>70 years) with stage II colon cancer had no survival benefit from adjuvant chemotherapy. - ctDNA analysis guides adjuvant therapy decisions but is limited by low analytical sensitivity in low-risk stage II disease.	[17,45,46]
**Colorectal Cancer (Stage III)**	Shortening adjuvant chemotherapy duration for low-risk patients	No	- For low-risk stage III patients (T1–T3, N1), 3 months of CAPOX was as effective as 6 months in disease-free survival. - High-risk patients (T4, N2) still benefit from 6 months.	[11,14,17]
**Rectal Cancer**	Avoiding preoperative chemoradiotherapy (CRT) for patients with locally advanced rectal cancer based on tumor response to neoadjuvant FOLFOX chemotherapy	No	- The PROSPECT trial showed that 91% of patients receiving neoadjuvant FOLFOX followed by surgery avoided preoperative CRT without compromising outcomes.	[47]
**Colorectal Cancer (Metastatic)**	De-escalation strategies: maintenance therapy, stop-and-go approaches, and drug holidays	No	- Maintenance therapy (cetuximab alone after induction) reduced toxic effects with non-inferior outcomes. - Stop-and-go strategies with intermittent oxaliplatin reduced neurotoxicity. - Drug holidays were safe under careful clinical and biological monitoring.	[18,48,49]
**Non-Small-Cell Lung Cancer**	Use of ctDNA to guide de-escalation of tyrosine kinase inhibitors (TKIs) Reduction in immune checkpoint blocker (CPI) doses and/or treatment durations	Yes (ctDNA)	- ctDNA-guided TKI reduction or discontinuation prevented disease progression while reducing side effects. - In patients treated with CPIs, the reduction in the treatment dose and duration maintained efficacy and reduced toxicity.	[19,20,21]
**Head and Neck Cancer**	De-intensification strategies (e.g., reducing chemotherapy, radiotherapy)	Yes (multifactorial model)	- The NRG-HN005 trial demonstrated that de-intensification for HPV-positive patients was feasible based on risk models. - Identifying low-risk patients can guide safe de-escalation.	[23,24]
**Ovarian Cancer**	Use of PARP inhibitors and reduction in adjuvant chemotherapy intensity	Yes (BRCA mutation)	- Properly selected BRCA-mutated patients could benefit from reduced chemotherapy after surgery while maintaining oncologic control. - Trials are ongoing to assess feasibility and safety.	[50]
**Prostate Cancer**	Adjusting hormonal therapy intensity Exploring omics tools for individualized treatment	Yes (omics)	- Triplet therapy benefits all patients but may not be necessary for all metastatic cases. - Omics tools could refine de-escalation strategies.	[26,28,29]

## Data Availability

Not applicable. There is no data or materials involved in this review.
